# Evaluation of prices, availability and affordability of essential medicines in Lahore Division, Pakistan: A cross-sectional survey using WHO/HAI methodology

**DOI:** 10.1371/journal.pone.0216122

**Published:** 2019-04-25

**Authors:** Amna Saeed, Hamid Saeed, Zikria Saleem, Yu Fang, Zaheer-Ud-Din Babar

**Affiliations:** 1 University College of Pharmacy, University of the Punjab, Lahore, Pakistan; 2 Department of Pharmacy Administration and Clinical Pharmacy, School of Pharmacy, Xi’an Jiaotong University, Shaanxi, China; 3 Center for Drug Safety and Policy Research, Xian Jiaotong University, Xi’an, Shaanxi, China; 4 The Global Health Institute, Xi’an Jiaotong University, Shaanxi, China; 5 Shaanxi Centre for Health Reform and Development Research, Shaanxi, China; 6 Department of Pharmacy, School of Applied Sciences, University of Huddersfield, Huddersfield, West Yorkshire, United Kingdom; 7 School of Pharmacy, Faculty of Medical and Health Sciences, University of Auckland, Auckland, New Zealand; Instituto Rene Rachou, BRAZIL

## Abstract

Inadequate access to medicines affected by un-controlled prices is a major concern in developing countries, including Pakistan, which lacks comprehensive data on medicine prices. Thus, the objective of this study was to evaluate the prices, availability and affordability of essential medicines in Lahore division, Pakistan. The survey was undertaken from November, 2016 till March, 2017 by including 50 medicines, 14 from the WHO/HAI core list and 36 supplementary medicines from national essential medicine list (NEML) at public (n = 16) and private (n = 16) health facilities. The prices, availability and affordability of selected medicines were measured using a variant of the WHO/HAI standard methodology available on HAI website and WHO/HAI manual. A questionnaire was used for data collection from Lahore division. The prices were compared to International reference prices (IRPs) and the daily wage of a lowest paid unskilled government worker was used to calculate medicine affordability. Data suggested poor availability of originator brands (OB) in public and private sector facilities, i.e., 6.8% and 55.0%, respectively. Similarly, low availability was observed for lowest price generics (LPGs), both in public (35.3%) and private sector (20.3%) facilities–far below the WHO global action plan targets of 80% availability of essential medicines by 2025. In private sector, 53% OB and 38% LPG medicines were found excessively priced. The cost of standard treatment with OBs was unaffordable, i.e., above a single daily wage (1.4 day’s wages) was demanded to purchase the standard treatment for the selected diseases in case of OBs medicines. Whereas, the cost of LPGs medicine required to purchase the standard treatment of the selected diseases was 0.6 day’s wage (median), below a single daily wage. In conclusion, access to essential medicines, especially at public sector facilities was affected by low availability, particularly of OBs in comparison to LPGs. Thus, the better availability of LPGs might be a rational basis of transition into a generic system of prescribing that may improve the availability and accessibility of essential medicines in Lahore division. Medicine prices in Lahore division were found higher in comparison to IRPs. Thus, the efforts must be made to formulate patient’s pocket friendly drug pricing policy that favors price cuts and improves affordability.

## Introduction

In developing countries, the budget allocated to the purchase of medicines contributes significantly to the overall health care costs and may account for almost 50–95% non-personnel costs [1]. In Pakistan, 32% of the health expenditure is borne by public sector, while 64% is borne by patient’s out of pocket payments that may account for unaffordability of medicines in this region [2]. Thus, the higher prices of medicines are considered as one of the major barriers to access medicines, even the essential medicines [2]. Whereas, availability and affordability are considered to be the key prerequisites for universal access to medicines [3].

Therefore, the estimation and comparison of medicine prices affecting medicines availability and affordability would be instrumental in framing a rational drugs pricing policy. A very few studies have been reported from Pakistan that deal with the ever-growing issue of increased/higher drug prices which may impact the affordability and availability of drugs [4]. Thirteen percent (13%) of Pakistan’s population lives below the poverty line, i.e. less than 1USD per day, the earning that is not sufficient to obtain even basic medicines such as aspirin for long term use [5].

In this context, the ministry of health, government of Pakistan had been providing free medicines in the public sector hospitals, nevertheless, the poor availability of medicines in public health facilities might compel the patients to get their medicines from private sector medicine retail outlets where they are supposed to pay from their own pockets [6]. To improve this situation, Drug Regulatory Authority of Pakistan (DRAP), after its formation in 2012, introduced its first ever drug price policy in 2015 to ensure transparent mechanism of price fixation in a bid to improve the availability and affordability of medicines [7]. As a control measure, annual increase in medicine prices was linked with Consumer Price Index (CPI), announced by Pakistan Bureau of Statistics, Government of Pakistan, with an allowable price increase of 4% for schedule drugs and 6% for non-schedule drugs. A few reports from Pakistan published in 2014 and 2016 claimed that the medicine prices were increased up to 100% [8–10], which was justified by pharmaceutical manufacturers mainly due to increase in operational costs associated with depreciation of Pak rupees against US dollar. Pakistan utilizes external reference pricing system by taking into account medicine prices of other countries as reference to set and control its medicine prices, which include, India, Bangladesh, New Zealand and United Kingdom, yet the prices are high and unaffordable. [10].

Very few studies have been reported on Pakistan’s Pharmaceutical pricing, in fact, there is only one study published in 2006 on availability and affordability of essential/medicines [6]. Therefore, there is an urgent need to evaluate the prices, availability and affordability of essential medicines in this region at different levels of health care. Thus, the objective of this study was to determine the prices, availability and affordability of originator brands and generic equivalents of the selected essential medicines in both public and private sector health facilities of the most populous division of Pakistan, the Lahore division. The study also compared these prices with international reference prices of selected countries of the region.

## Methodology

### Study design

**Fig 1:**
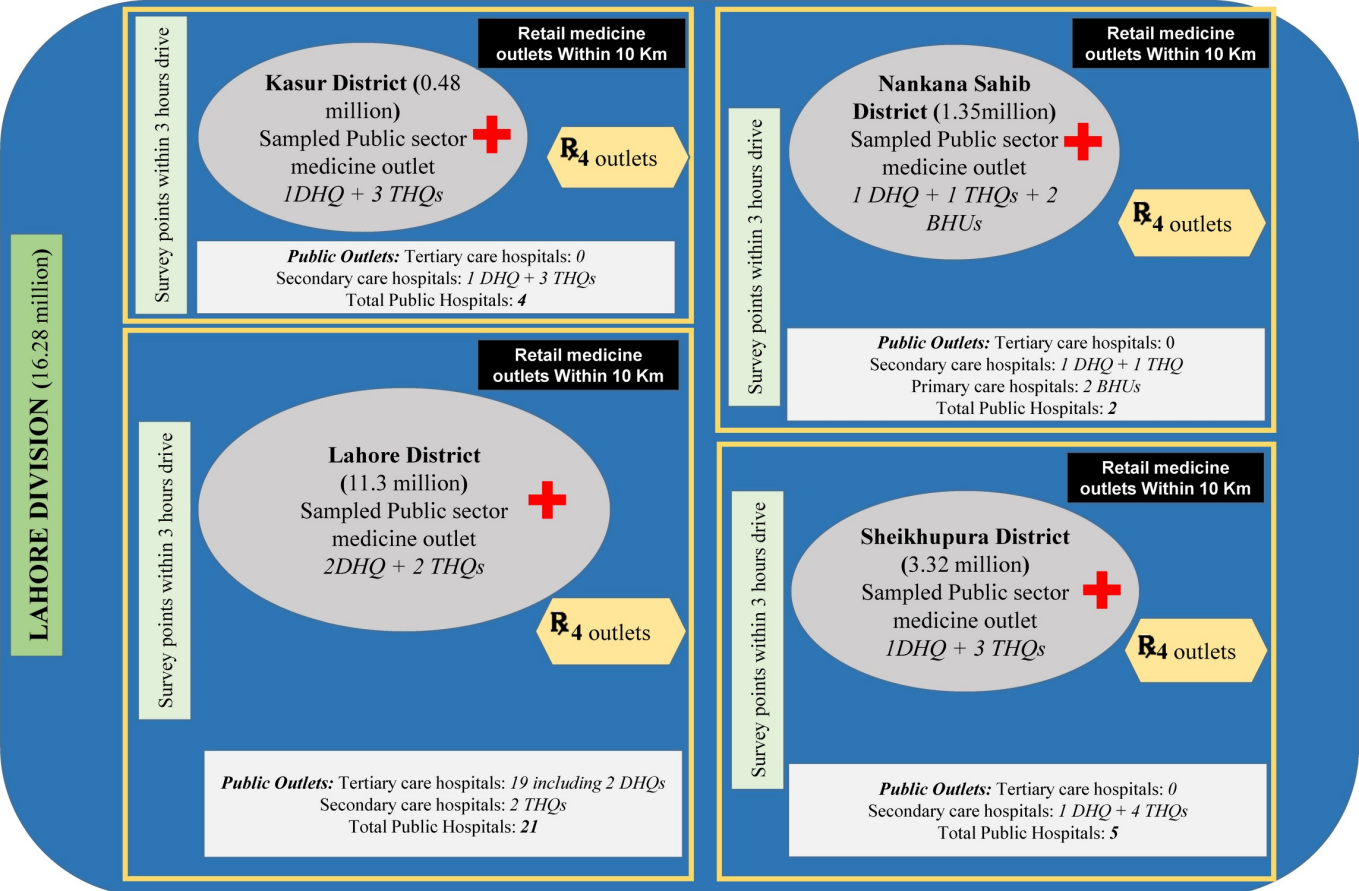
Macrograph depicting sampling sectors of the study along with district wise distribution of population, total hospitals and sampled public sector medicine outlets. **DHQ;** district head quarter hospital, **THQ;** tehsil head quarter hospital and **BHU;** basic health unit. In Lahore district, 2 Tertiary care hospitals are given the status of DHQ, one of these DHQs was taken as survey anchor. The Red Cross symbol indicates a public sector medicine outlet and the prescription symbol indicates a private sector medicine outlet.

A cross-sectional study was conducted by employing a variant of World Health Organization (WHO) /Health Action International (HAI) methodology in Lahore Division, Pakistan [11]. Data on medicine prices, availability and affordability for specific list of medicines was collected from four districts of Lahore division, i.e., Lahore, Kasur, Nankana Sahib and Sheikhupura (Fig 1). The sampled health facilities were surveyed from November 2016 to March 2017 (referring to the fiscal year 2016–17, as the annual budget is announced in June by the government of Pakistan).

### Survey region

Pakistan consists of four provinces which are sub-divided into several administrative units called “divisions”, each division is further sub-divided into districts, then districts into tehsils and tehsils into union councils. The survey region, Lahore division, is situated within the largest province of Pakistan (in terms of population), i.e. Punjab, which consists of four districts with a population of 16.28 million as of 2017 [12]. According to standard WHO/HAI methodology, six regions must be selected as “survey areas”—the administrative units (e.g. provinces, divisions, cities, districts or countries) with estimated population coverage of about 0.1 to 0.25 million. All survey areas (e.g. four districts in our case) must be reachable in a day from the main urban center (e.g. Lahore district in our case). However, we customized the methodology and used a variant of the standard methodology because Lahore Division consisted of four administrative divisions/districts, thus instead of six survey areas, four regions were selected as survey areas [11]. Moreover, the number of NGO sector facilities were very few to be considered for analysis, none in Kasur, Sheikhupura and Nankana Sahib, (because the number of these sector facilities should be four according to the WHO/HAI methodology), thus NGO sector facilities were not included in the survey.

### Sampling of medicine outlets for survey

Data were collected from a sample of public sector medicine outlets and from registered retail pharmacies (private sector) in Lahore division. According to the standard methodology of WHO/HAI, 4 public sector outlets must be selected from each survey area. In public health sector of Pakistan, tertiary care services are provided by teaching hospitals, secondary healthcare services are usually provided by district head quarter (DHQs) and tehsil head quarter (THQs) hospitals, while basic healthcare units (BHUs) provide primary level of healthcare. But there is one exception in case of Lahore district (a larger district as compared to other three districts in the study) i.e. two tertiary care hospitals are given the status of DHQs by the ministry of health. The DHQs are located in main cities whereas THQs and BHUs are located in peripheral cities/towns.

The standard methodology suggests to include the main public sector hospital from each region as survey anchor (DHQs in our case) and to select other public sector outlets randomly which are within 3 hours travel from the survey anchor. Since Lahore division is divided into 4 districts, therefore, we selected a total of 16 public sector hospitals, 4 from each district (Fig 1).

- From Lahore district, all 4 available DHQs and THQs were selected i.e. 2 DHQs (tertiary care hospitals) and 2 THQs (secondary care hospitals).
- In district Kasur, where 4 secondary healthcare public sector hospitals are available, which include 1 DHQ and 3 THQs, all of these where surveyed.
- In Sheikhupura district, where total 5 secondary healthcare hospitals are available, which include 1 DHQ and 4 THQs, after selecting the DHQ as survey anchor, we selected 3 THQs randomly to complete the total of 4 hospitals in this survey region.
- In Nanakana Sahib district, there were two secondary healthcare hospitals. Thus, because of the lesser number of THQs in Nankana as compared to other three districts, only option left was to select 2 BHUs located within 3hrs drive from the main public hospital (i.e. DHQ) to complete 4 public sector outlets in this region (Fig 1).

Overall, 14 out of 32 public sector tertiary and secondary care hospitals were surveyed along with 2 BHUs as public sector medicine outlets. (Fig 1).

From private sector, retail pharmacies were selected as per WHO/HAI methodology. The criteria suggests to include one private pharmacy outlet within 10 Km range of each public sector health facility (Fig 1). In this way, a total of 16 private retail pharmacies were selected which included 1 private retail pharmacy near to each selected public sector medicine outlet. It is also noteworthy that each survey unit (which includes one public and one private sector medicine outlets) was located in different tehsils (administrative sub-division) of a particular district. Thus, out of 17 tehsils in Lahore division, 16 tehsils were included in the survey ensuring fair representation of the division Lahore. As a result, 32 healthcare facilities/medicine outlets were surveyed in the study.

### Medicine selection

According to WHO/HAI methodology, the selection of medicine for the surveys should include core and supplementary list of medicines selected by each country based on local needs and disease burden [11]. The list of survey medicines included 14 core essential medicines from WHO/HAI core list for local and international comparison [11] as suggested by WHO/HAI methodology standards. Medicines with specific formulation, dosage forms, and procured via donations were not included in the study taking into account their infrequent usage for specific diseases and procedures, non-enlistment in the NEML and difficulty in price estimation of donated medicines. The survey also included 36 supplementary medicines, selected based on local population disease needs, by taking into account WHO/HAI methodology standards. Therefore, most of the medicines were chosen from Pakistan’s national essential medicines list (NEML) 2016 considering local disease burden. Thus, out of 50 medicines, 46 surveyed medicines were part of Pakistan’s NEML 2016, Table A in S1 Text [8].

### Data collection and analysis

Data were collected by trained data collectors (Pharmacy students) who visited the retail medicine outlets and recorded the availability and prices of the selected medicines using a data collection form (Appendix 1). The form was used to get the required drug pricing information from each outlet. The availability of each medicine was marked after physical checking and proper documentation. The prices of originator brands (OBs), the original patented pharmaceutical products, and lowest price generics (LPGs) available in the facility at the time of survey were collected from 16 public and 16 private medicine outlets.

Data were entered in Excel Workbook which is available on HAI website along with the WHO/HAI manual [11]. Availability, median price ratios and affordability of selected medicines were calculated according to the standard WHO/HAI methodology. All prices were converted into US dollars using the exchange rate from OANDA (Canada) currency converter on November 05, 2016, the first day of survey i.e. 1USD = 103.7020 PKR [13].

#### Medicines availability

Availability of each medicine was documented as percent availability of surveyed medicines in a facility on the day of data collection [11]. Availability was documented as follows; Absent: 0% of facilities, these medicines were not found in any facility; Low: < 50% of facilities, these medicines were rarely found; Fairly high: 50–80% of facilities, these medicines were found at several facilities; High: > 80% of facilities, these medicines were found in most of the facilities. [14].

#### International reference price and median price ratios

Medicines prices were calculated in terms of median price ratios (MPRs). Median Price Ratio is the ratio of median local unit price obtained during the survey to the international reference unit price, estimated mainly to facilitate international price comparisons. MPR was obtained using the formula given below;

Median Price Ratio (MPR) = Median local unit price **/** International reference unit price

Employing MPRs, medicines can be categorized as high or low-cost medicines—higher MPR means higher price. MPR of more than 5 for private sector and more than 2 for public sector has been considered as cut off values for fairly high prices by many studies [14]. Whereas, the MPR of greater than 2 indicates that the local medicine prices were twice compared to international reference prices. Still, there are no well-defined rules for the interpretation of MPRs because number of key medicine price components vary among different countries, such as different market size and penetration, medicines pricing mechanisms, accessibility, taxation and scales of economy. So, we took an MPR of more than 1 for public sector and MPR of more than 2 for private sector as a cut off value for excessively priced medicines, as this criterion has already been used by previous study conducted in Pakistan using similar methodology [6]. WHO & HAI recommend the use of Management Sciences for Health (MSH) International Drug Price Indicator Guide as the source of reference prices. In this study, Price Indicator Guide 2015 was used as a source of international reference prices of the survey medicines. The MSH prices are net prices mostly obtained from non-profit suppliers to the developing countries and NGOs. In this study, the median unit supplier prices were used. These MSH reference prices are relatively low and offer a very useful standard to compare locally available medicines [15].

#### Affordability

Medicine affordability was estimated by considering the number of working days (daily wages) of the lowest paid unskilled government employee that enable him/her to purchase the course of standard treatment for common conditions with selected medicines. If in a month, a patient spends more than one day’s wage for getting the standard treatment then it was considered unaffordable [11]. Patient’s affordability of standard treatments for different diseases was calculated by taking into account the salary of lowest paid government worker for the fiscal year 2016–17, 14000 Pak Rupees/month (with effect from 01 July 2016) [16]. A total of 12 medicines were considered for affordability calculation, including medicines from WHO core list used for most common chronic diseases, such as cardiovascular diseases and diabetes. In case of medicines for chronic diseases, total treatment duration of 30 days was considered for affordability calculations, since the affordability was estimated for a month. The number of units of selected medicines for a standard treatment and total duration of therapy were entered into the WHO/HAI preprogrammed Excel workbook as suggested by WHO/HAI methodology. Both the median treatment price (in local currency) and the number of daily wages spent on the treatment were automatically calculated for both private and public sectors. Treatment that cost only 1 day income or less (1 month supply in case of chronic conditions) was considered affordable.

### Ethics statement

Ethical approval of the study was obtained from ethics committee on human research, University College of Pharmacy, University of the Punjab, Lahore, Pakistan with reference # EC/PUCP/193/2017. Patients and public were not involved in this study. A formal application from principal investigator along with approval letter from University was submitted to hospital medical superintendent to get permission for data collection from each health facility.

## Results

### Availability of medicines in public and private sector

Data on the availability of medicines in public and private sector facilities are summarized in Table 1. We found that the overall availability of surveyed medicines in public sector was 6.8% only (mean percent availability) for originator brands (OBs) and 35.3% (mean percent availability) for the lowest price generics (LPGs) (Table 1). Compared to public sector, the availability of medicines in private sector retail pharmacies for 50 surveyed medicines was relatively better in case of OBs i.e. 55% and relatively lower in case of LPGs i.e. 20.3%, Table B in S1 Text.

**Table 1:** Individual medicines availability in outlets.

Medicine Name	OBs (%)		LPGs (%)	
	Public (n = 16)	Private (n = 16)	Public (n = 16)	Private (n = 16)
**Acetylsalicylic Acid**	0.0	25.0	56.3	25.0
**Aciclovir**	0.0	50.0	25.0	37.5
**Amiodarone**	9.1	62.5	18.2	6.3
**Amitriptyline**	0.0	31.3	0.0	25.0
**Amlodipine**	6.3	75.0	62.5	31.3
**Amoxicillin**	43.8	93.8	18.8	25.0
**Amoxicillin (250)**	18.8	100.0	12.5	31.3
**Atenolol**	12.5	87.5	81.3	43.8
**Atorvastatin**	9.1	50.0	18.2	25.0
**Azithromycin**	0.0	25.0	25.0	31.3
**Beclometasone inhaler**	0.0	6.3	36.4	12.5
**Bisoprolol**	6.3	87.5	18.8	25.0
**Captopril**	0.0	81.3	81.8	25.0
**Carbamazepine**	12.5	87.5	56.3	25.0
**Ceftriaxone injection**	18.2	68.8	81.8	31.3
**Ciprofloxacin**	0.0	81.3	100.0	37.5
**Clarithromycin**	0.0	87.5	45.5	25.0
**Co-trimoxazole suspension**	6.3	31.3	62.5	6.3
**Diazepam**	6.3	62.5	12.5	0.0
**Diclofenac**	54.5	50.0	36.4	6.3
**Digoxin**	0.0	62.5	36.4	0.0
**Enalapril**	0.0	68.8	62.5	18.8
**Fluconazole**	0.0	37.5	18.8	25.0
**Fluoxetine**	0.0	43.8	12.5	43.8
**Fluphenazine Decanoate**	0.0	0.0	0.0	12.5
**Furosemide**	12.5	93.8	50.0	6.3
**Glibenclamide**	18.8	68.8	56.3	12.5
**Gliclazide**	0.0	75.0	0.0	18.8
**Hydrochlorothiazide**	0.0	0.0	0.0	12.5
**Indinavir**	0.0	0.0	0.0	0.0
**Insulin Isophane (NPH)**	0.0	75.0	50.0	25.0
**Insulin Neutral Soluble (Regular)**	0.0	68.8	50.0	25.0
**Losartan**	9.1	43.8	18.2	43.8
**Lovastatin**	0.0	0.0	6.3	0.0
**Metformin**	25.0	81.3	56.3	31.3
**Methyldopa**	12.5	81.3	25.0	0.0
**Metronidazole**	25.0	93.8	56.3	37.5
**Nevirapine**	0.0	0.0	9.1	0.0
**Nifedipine Retard**	0.0	75.0	18.2	12.5
**Omeprazole**	6.3	56.3	75.0	37.5
**Omeprazole (10)**	0.0	12.5	43.8	18.8
**Paracetamol suspension**	6.3	68.8	87.5	25.0
**Phenytoin**	0.0	6.3	12.5	6.3
**Propranolol**	12.5	68.8	12.5	6.3
**Pyrimethamine with sulfadoxine**	0.0	75.0	18.2	6.3
**Ranitidine**	0.0	81.3	31.3	25.0
**Salbutamol inhaler**	0.0	81.3	56.3	31.3
**Simvastatin**	0.0	25.0	27.3	56.3
**Spironolactone**	6.3	62.5	25.0	0.0
**Zidovudine**	0.0	0.0	0.0	0.0

OBs: Originator brands

LPGs: Lowest priced generics

In public sector, the mean availability of LPGs was 35.3%, while it was 20.3% in private sector facilities, Table B in S1 Text. In public sector, only 5 out of 50 LPG medicines, surveyed in the study had adequate availability, i.e. 80%, while 6 medicines—Amitriptyline, Fluphenazine Decanoate, Gliclazide, Hydrochlorothiazide, Indinavir, Zidovudine were not available at any facility. The overall availability of OBs in public sector was as low as 6.8% in public sector while it was 55% in private sector facilities. Moreover, in more than 75% public sector facilities not even a single originator brand was available, Table C in S1 Text. In private sector, the mean availability of all and essential medicines was 55% for OBs and 20.3% for LPGs. Four medicines, i-e., Indinavir, Lovastatin, Nevirapine and Zidovudine (LPGs and OBs), were absolutely not available in all selected outlets (both public and private sectors), Table C in S1 Text. Likewise, out of all the surveyed LPG medicines, eight medicines were not available at any outlet, including Diazepam, Digoxin, Lovastatin, Methyldopa, Spironolactone, Nevirapine, Indinavir and Zidovudine. The medicines with availability of more than 80% were Atenolol, Captopril, Ceftriaxone Injection, Amoxicillin 250mg and Ciprofloxacin, Table D in S1 Text.

### Medicines availability at various health care levels

In Public sector, availability of medicines at particular level of care i.e. primary, secondary and tertiary healthcare units, was also estimated and only those medicines were included for calculation which were supposed to be available at every level of care according to Pakistan’s NEML. At primary level, of all the medicines, not a single OB was available, while only 25.8% LPG medicines were available, Table C in S1 Text. At secondary health care level, availability of medicines improved in comparison to primary level, i-e., 7.1% for OB and 35.2% for LPG medicines. At tertiary level, the availability of medicines was even better than the primary and secondary health care levels, i-e., 12% for OB and 50% for LPG medicines, Table C in S1 Text. However, ideally the percent availability should be more than 80% as defined by the standard WHO/HAI methodology, but none of the healthcare facilities, irrespective of the type, under this analysis fulfills that criterion.

### Patient prices in private sector

**Fig 2:**
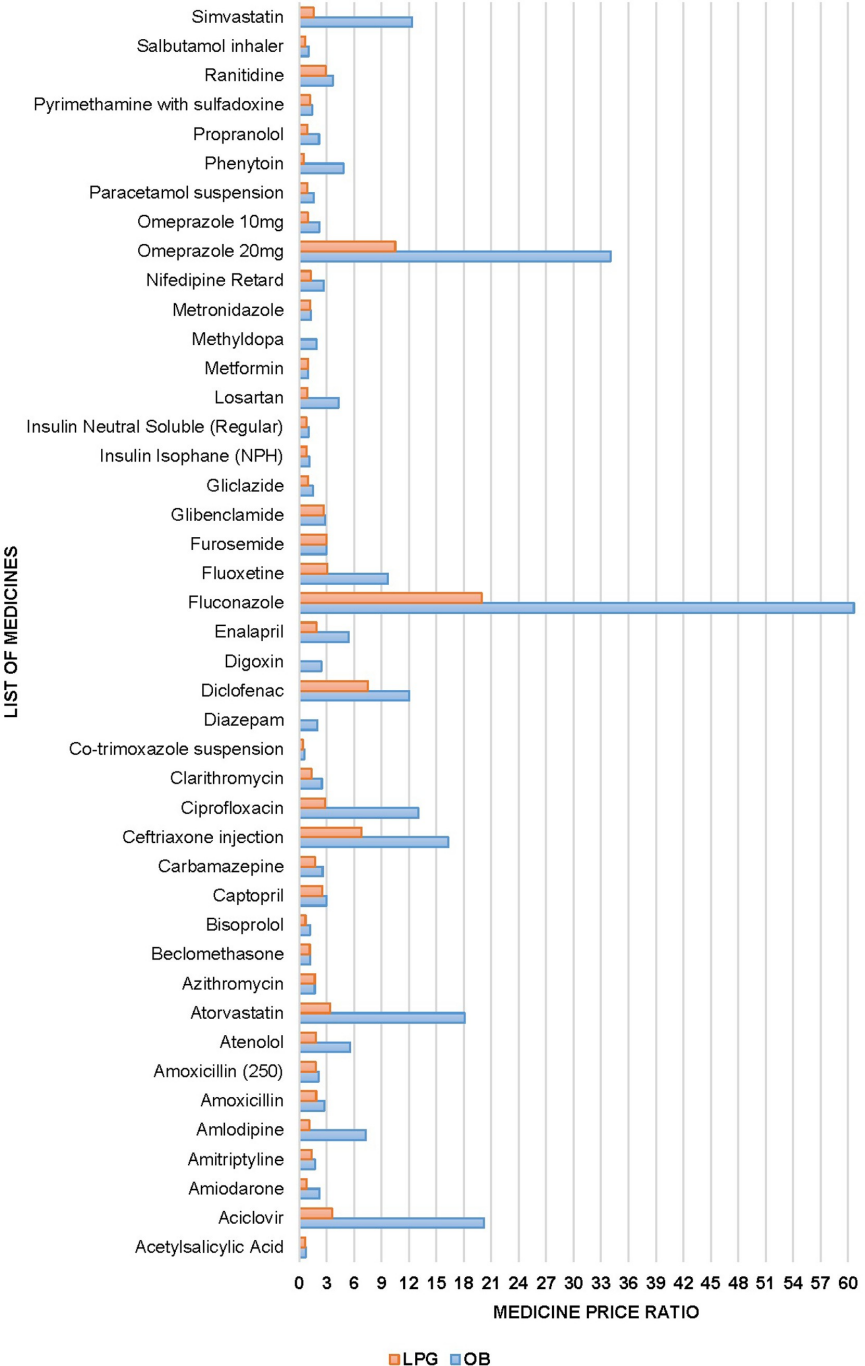
Individual medicine price ratios for surveyed medicines in private sector.

In public sector heath facilities, patient prices i.e. the prices paid by patients to get the medicines, were not estimated due to the provision of free medicines. Data revealed that in private sector overall MPRs for all the surveyed medicines, compared to MSH (management science health) 2015 reference prices, varied between 0.42 and 60.63 i.e. the prices in Pakistan were higher, somewhat between 2.5 times lower to 60 times higher, than the international reference prices, Table E in S1 Text. The price variations were higher for OB products, i-e., between 0.58 and 60.63 in terms of median MPR, while the variations were lower for LPG medicines, i.e. 0.42 to 19.96. Additionally, almost 53% OBs and 38% LPGs fell in high priced medicine category i.e. having MPR of more than 2, Table E in S1 Text. The top five OBs with highest MPRs include, Fluconazole (60.63), Omeprazole 20mg (34.04), Aciclovir (20.21), Atorvastatin (18.11) and Ceftriaxone inj. (16.28). The top five LPGs with highest MPRs, include Fluconazole (19.96), Omeprazole 20mg (10.50), Diclofenac (7.50), Ceftriaxone inj. (7.50) and Atorvastatin (3.36) (Fig 2).

### Affordability of standard treatments at private facilities

On an average, the cost of standard treatment was equivalent to 1.4 day’s wages (median) for OBs and 0.6 (median) for LPG medicines (Table 2). Notable treatments exceeding the minimum daily wage limits for OBs included, Simvastatin (4.3), Omeprazole (3.2), Bisoprolol (1.5), Ciprofloxacin (1.5), Insulin Isophane (NPH) (1.4) and Insulin Neutral Soluble (Regular) (1.4)–all were considered unaffordable (Table 2). However, the treatment with LPG medicines seems affordable and were either equal or less than one day’s wage, though with poor availability (20.7%) (Table 2). It is pertinent to mention that this calculation was done by taking into account the standard dose of individual medicines, if a patient is taking more than one medicines then the bar will go even higher making the treatment completely out of reach for most of the patients.

**Table 2:** Affordability of standard treatments in private retail pharmacies by lowest paid unskilled government worker.

	Disease Condition and Standard Treatment				No. of units needed per treatment	Duration of days	Median treatment price (in Pak rupees)		Day’s Wages for Treatment	
Sr. no.	Disease/ Condition	Medicine	Strength	Dosage form			OB	LPG	OB	LPG
**1**	Asthma	Salbutamol Inhaler	100mcg/dose	Inhaler	200	As needed	200	128	0.4	0.3
**2**	Cardiovascular Diseases	Bisoprolol	5mg	tab	60	30	676.8	393	1.5	0.8
**3**		Captopril	25mg	tab	60	30	459.6	390	1.0	0.8
**4**	Hypercholesterolemia	Simvastatin	20mg	Cap/tab	30	30	2013.9	255	4.3	0.5
**5**	Adult Respiratory Infection	Ceftriaxone Injection	1g/vial	Inj.	1	1	672	280	1.4	0.6
**6**		Ciprofloxacin	500mg	tab	14	7	705.6	154	1.5	0.3
**7**	Fungal Infection	Fluconazole	200mg	cap	1	1	442	145.5	0.9	0.3
**8**	Epilepsy	Carbamezipine	200mg	tab	60	30	294.6	198	0.6	0.4
**9**	Diabetes	Glibenclamide	5mg	tab	90	30	152	141	0.3	0.0
**10**		Insulin Isophane (NPH)	100IU/ml	vial	10^#^	30	645	480	1.4	1.0
**11**		Insulin Neutral Soluble (Regular)	100IU/ml	vial	10^#^	30	645	480	1.4	1.0
**12**	Ulcer	Omeprazole	20mg	cap	30	30	1493.40	460.5	3.2	1.0

#each unit considered as milliliter(ml).

## Discussion

The present study clearly demonstrated that both OBs and LPGs of the surveyed essential medicines were excessively priced in private sector pharmacies in Lahore Division, the most populous division of Pakistan. Moreover, the present survey was carried out over an extended period of time (i.e.4-5 months) at different health facilities, therefore, it provided a realistic assessment of the overall situation faced by the patients on daily basis. The overall availability of OBs was extremely poor in public sector medicine outlets in comparison to private sector medicine outlets, while for LPGs it was better compared to OBs, but still very skimpy in both sectors. Similarly, the treatment of common diseases was found affordable with LPGs but unaffordable with OBs. When we compared our data with other middle-income countries (Egypt, India, Lebanon and China), Pakistan was positioned at number three, after India and Lebanon, in terms of medicine’s affordability, though these differences seemed trivial due to small sample size.

Availability and affordability are considered the key requisites for universal access to medicine [3, 17, 18]. In 2004, 12–13 years back, a study from Pakistan reported that OBs were not available in public sector and only 3.3% (median percent availability) LPGs were available at public sector facilities [6]. Despite divisional/regional focus of our study, data of the current study was compared with nation wide study on medicine prices, since the medicines in Pakistan are priced by central government and are supposed to be supplied and sold at similar prices across the country.

In this regard, compared to the situation in 2004, the data from the present study suggested that the overall availability of surveyed medicines in public sector exhibited improvements—significant in case of LPGs (35.3%) and fairly inadequate in case of OBs (6.8%) [6]. Probably, these improvements are attributable to the establishment of DRAP in 2012 which resulted in the enforcement and procurement of NEML based system of medicines coupled with increase in the number of working pharmacists in public sector facilities due to surge in hiring. However, DRAP’s bid to exercise the statutory powers in order to regulate the prices of medicines were opposed by manufacturers on the premise of having poor revenues due to limited whole sale markups, i-e., 2 to 10%. Thus, in spite of DRAP’s approval, between June–August, 2016, to increase the prices 4 times, many companies increased up to 5 times and were adamant on upholding their decision by getting stay orders from Lahore High Court. Notwithstanding the improvements in drug regulation, the availability of medicines was still below the optimal benchmark, i.e. 80%, in Lahore division, Pakistan. However, when compared with primary and secondary health care facilities, the availability of medicines was relatively better at a tertiary care facility of Lahore, but still below the WHO touchstone of 80% regarding availability of essential medicines.

Data further suggested that compared to public sector, the availability of surveyed medicines was higher in private sector. While in 2004, the availability of medicines in private sector was reported as 54.2% for OBs and 31.3% for LPGs, which has now been improved to 55.0% in case of OBs and considerably reduced to 20.3% in case of LPGs [6]. The reduced availability of LPGs in recent year suggested that the patients are compelled to purchase the expensive medicines in private sector due to non-availability of cheaper medicines compared to previous years. Despite the enforcement of NEML based procurement system in public sector hospitals and implementation of DRAP pricing policy in 2016, it became practically non-viable for many pharmaceutical companies, including multinationals, to manufacture pharmaceuticals or sell them to public funded hospitals at cheaper prices. Seemingly, the major reason could be the unceasing stalemate between pharma companies and DRAP on price regulation, which may have resulted into stock outs of essential medicines, especially in public funded hospitals, affecting availability. Therefore, it is reasonable to assume that the majority of Pakistan’s population have poor access to cheaper medicines (LPGs), simply because most of the patients, i-e., 67% or more consult private physician and often seek treatment from a private pharmacy rather than a basic or rural health unit [6]. Other reasons for poor availability of LPGs could be the budgetary constraints, poor regulation of medicines in terms of procurement, insufficient NEML driven procurement policies (to purchase lowest price medicine), fragile supply chain and non-functional pharmacy and therapeutics committees.

It has been reported previously that the overall MPRs in private sector of Pakistan varied from 0.20 to 26.20 in 2004, i.e., from 5 times lower to 26 times higher prices as listed by MSH[6]. Data from the present study suggested that MPRs in private sector exhibited significant variation from 4 times lower to 60 times higher for all the surveyed medicines taking into account MSH 2015 reference prices. Additionally, of all the medicines included in the survey, 53% of OBs and 38% of LPGs were found overpriced i.e. having MPR of more than 2. Despite the establishment of DRAP and more comprehensive drug pricing policy in Pakistan, the increase in medicine prices over the past decade could be due to several factors, such as poor implementation and regulation by DRAP, devaluation of Pakistani rupee, lack of entry agreement, value-based pricing, intended benefits to importers and multinational pharma companies, and imbalance between affordability and profitability. The better availability of OBs in private sector might be attributed to frequent prescriptions of brands by the doctors and incentive driven purchase/stocking of these brands at pharmacies by the pharmacists, which possibly affect the availability of LPGs in private sector.

In Pakistan, approximately one third of the population lives below the national poverty line and 36.9 million out of 185 million people live with or earn less than 325.05 Rupees per day–less than the daily wage affordability calculation i.e. 466.6 Rupees [19]. This clearly indicates that basic essential treatments for the prevalent chronic diseases will be completely out of reach for millions of Pakistanis. Therefore, the generic prescribing must be mandated at least in public sector. The doctors must be trained to work out the cost-effective therapy for the patients by emphasizing generic prescribing. Seminars and workshops should be arranged for pharmacists and medicines procurement officers to train them in the efficient management of supply chains.

There could be several implications for policy makers that need to be accentuated while devising medicine pricing policy. The government should ensure and evaluate NEML based procurement which must be revised on regular basis. Innovative financing mechanisms that support the sale of a group of essential medicines in private sector retail pharmacies. There should be more efficient procurement system duly supported by sustainable financing to improve the availability of essential medicines at public medicine outlets. Moreover, drug regulators in collaboration with health department should ensure focused resource utilization on selected generic essential disease medicines instead of broad range of originator brand and local generic medicines to ensure improved availability of priority treatments. Similarly, lowering procurement prices, promoting and implementing differential pricing for countries like Pakistan, exempting essential medicines from tariffs and taxes, and promoting local manufacturing of essential brands by subsidizing raw material purchase would improve essential medicine availability and affordability for the sick. Combining together, the above stated recommendations along with consumer awareness campaigns about medicine prices and encouraging regressive markups rather than progressive markups on costly medicines can be instrumental in bringing down the prices of medicines.

Thus, the poor availability of essential medicines in the public sector of Pakistan can be translated into limited or no access to medicines for the poor. Probably, the poor adherence to pricing policy is also one of the major reasons of failure to control prices of drugs and inaccessibility of essential medicines to the people of Pakistan. Moreover, many retailers are overpricing the drugs and are not selling them on the maximum retail prices fixed by the drug pricing committee [6]. So, a system should be devised to regularly monitor the MRPs of medicines in private sector and to ensure adherence to pricing policy. These assessments also necessitate such comparisons to be done at a larger scale.

### International comparison of affordability of standard treatment

A total of 4 middle income countries, i.e. India, Egypt (lower middle income countries), China & Lebanon (Upper middle income countries) were selected to compare the affordability with that of Pakistan [20]. India resides in the South Asian region—Pakistan’s neighboring country having almost similar medicine pricing and procurement systems. Pakistan also considers India’s medicine pricing as an external reference for its own medicine pricing. Pakistan is also a member of WHO Eastern Mediterranean Region which include Egypt and Lebanon [21]. China borders Pakistan’s northeast side and falls under the category of upper middle income country by World Bank [20]. Another reason for selecting these particular countries was the availability of pricing data from studies conducted in these countries using similar methodology, at HAI’s database of medicines pricing. Latest available surveys were selected for comparison [22]. We evaluated the affordability of 12 originator brands in private sector of Pakistan and compared these with private sector of other middle-income countries i.e. India, China, Egypt, Lebanon, using the WHO/HAI medicine prices database [21–23]. It was found that the treatment for asthma, salbutamol inhaler, was more affordable in Egypt (0.3) compared to Pakistan (0.4) and other selected countries (Table 3). Likewise, for hypertension, captopril was more affordable for patients in Egypt (0.8) and China (0.6) compared to Pakistan (1.0). Insulin Isophane (NPH) and Insulin Neutral Soluble (Regular) were less affordable in Egypt (0.8) and even in upper middle-income country, i-e., China (1.1) in comparison to Pakistan (1.4). Affordability of majority of the medicines was comparable between India and Pakistan, nevertheless, the number of daily wages needed to get the standard treatment with originator brand of Simvastatin was found to be two times higher in Pakistan compared to India and Lebanon [22, 24]. But the number of daily wages spent on obtaining standard treatment with Omeprazole were almost three times higher in Pakistan compared to China. This data suggested that ulcer treatment remained non-affordable to a vast majority of the population in Pakistan, despite its growing prevalence. Overall, Lebanon was found to be the most affordable country in terms of obtaining standard treatment with selected medicines followed by India and Pakistan at second and third positions, respectively. The ranking has been done on the basis of average no. of days’ wages required to get the standard treatment with selected medicines. The lesser no. of days’ wages required, the more affordable would be the treatment in a particular country (Table 3). However, possibly due to small sampling, the differences appeared trivial.

**Table 3:** International comparison of affordability of standard treatments.

Condition/Disease & Medicine	Affordability (No. of day’s wages required)				
	Country and date of survey				
	Pakistan 2016–17	India (NCT-Delhi) July 2011	Egypt 2013	Lebanon July 2013	China Shaanxi Province-2014
**Asthma** Salbutamol Inhaler	0.4	0.4	0.3	0.4	0.60
**Cardiovascular Diseases** Captopril Atenolol	1.0	NA	0.8	1.3	0.60
	0.4	0.4	0.4	0.7	NA
**Hypercholesterolemia** Simvastatin	4.3	2.2	4.3	1.8	7.10
**Adult Respiratory Infection** Amoxicillin Ciprofloxacin	0.4	NA	NA	NA	1.40
	1.5	NA	NA	NA	0.40
**Depression** Amitriptyline	0.3	1.2	NA	0.4	0.70
**Diabetes** Glibenclamide Insulin Isophane (NPH) Insulin Neutral Soluble (Regular)	0.3	0.3	0.4	0.9	NA
	1.4	NA	0.8	1.1	NA
	1.4	NA	0.8	1.1	NA
**Ulcer** Omeprazole	3.2	NA	2.9	1.4	0.80
**Arthritis Pain/Inflammation** Diclofenac Paracetamol Suspension	0.7	0.8	NA	2	NA
	0.1	0.1	NA	NA	NA
**Average Affordability**	1.18	1.17	1.33	1.11	1.65

### Study limitations

The data on availability of medicines was collected at a specific point in time, thus may not reflect the average availability of medicines over the time. For sampling, district wise sampling of health facilities cannot be opted uniformly to ensure random selection, since not all districts had desired availability of tertiary and primary care health facilities, such as Lahore and Nankana sahib. Thus, sampling had to be relied upon available health facilities in each district. The affordability was estimated considering government lowest daily wage worker’s salary that might represent an over-estimation of affordability, since many private workers earn less than government daily wage worker. Moreover, except for a nationwide study conducted in 2004, not a single recent study was available from Pakistan particularly from Lahore division to compare the results of our study. Thus, the generalizability of the data to the whole country is questionable.

### Conclusion

Data revealed that in Lahore Division, Pakistan, the availability of OB medicines was extremely poor in public sector healthcare facilities in comparison to LPG medicines–yet the availability of both remain sub-optimal. In private sector medicine outlets, the availability of OB medicines was better compared to LPG medicines but with fairly higher prices impacting patient’s affordability. Moreover, essential LPG medicines were found affordable if consumed as sole drug therapy option, nevertheless, they become unaffordable if drug therapy includes more than one drug. Thus, several medicines, used in the treatment of common ailments such as hypertension, diabetes, ulcer and arthritis, were found unaffordable for majority population in Lahore Division, Pakistan. To improve the situation, key policy decisions should be implemented, such as use of generic medicines, sustainable and reliable procurement and financing, medicine price monitoring system, prevent excessive mark ups in the supply chain and continuing research into medicine pricing.

## Supporting information

S1 Text List of selected drugs, median price ratios, availability and affordability of medicines at each health care level and comparison of medicine MPRs, affordability to low and middle-income countries. (DOCX)
