# Supplementary material for: Evaluation of prices, availability and affordability of essential medicines in Lahore Division, Pakistan: A cross-sectional survey using WHO/HAI methodology
Source: PLoS One. 2019 Apr 25;14(4):e0216122. doi: 10.1371/journal.pone.0216122 (PMC6483245; doi:10.1371/journal.pone.0216122)
Supplement: S1 Text — (DOCX) [file pone.0216122.s001.docx]

**Table A.** List of Medicines Used in the Study.

| Sr. No. | Medicine Name  (Name must be unique) | Medicine Strength | Dosage Form | Medicine list | National Essential Medicine List | To be used at health service level* |
| --- | --- | --- | --- | --- | --- | --- |
| 1 | Acetylsalicylic Acid | 75mg | tab | Supplementary | Yes | 1 |
| 2 | Acyclovir | 200mg | tab | Supplementary | Yes | 1 |
| 3 | Amiodarone | 200mg | tab | Supplementary | Yes | 2 |
| 4 | Amitriptyline | 25 mg | cap/tab | Global | Yes | 3 |
| 5 | Amlodipine | 5mg | tab | Supplementary | Yes | 1 |
| 6 | Amoxicillin | 500 mg | cap/tab | Global | Yes | 1 |
| 7 | Amoxicillin (250) | 250 mg | cap/tab | Supplementary | Yes | 1 |
| 8 | Atenolol | 50mg | tab | Supplementary | Yes | 1 |
| 9 | Atorvastatin | 20mg | cap/tab | Supplementary | No | 2 |
| 10 | Azithromycin | 500mg | cap/tab | Supplementary | Yes | 1 |
| 11 | Beclometasone inhaler | 50 mcg/dose | dose | Supplementary | Yes | 2 |
| 12 | Bisoprolol | 5 mg | cap/tab | Global | Yes | 1 |
| 13 | Captopril | 25 mg | cap/tab | Global | No | 2 |
| 14 | Carbamazepine | 200 mg | cap/tab | Supplementary | Yes | 1 |
| 15 | Ceftriaxone injection | 1 g/vial | vial | Global | Yes | 2 |
| 16 | Ciprofloxacin | 500 mg | cap/tab | Global | Yes | 2 |
| 17 | Clarithromycin | 500mg | tab | Supplementary | Yes | 2 |
| 18 | Co-trimoxazole suspension | 8+40 mg/ml | millilitre | Global | Yes | 1 |
| 19 | Diazepam | 5 mg | cap/tab | Global | Yes | 1 |
| 20 | Diclofenac | 50mg | cap/tab | Global | Yes | 2 |
| 21 | Digoxin | 0.25mg | tab | Supplementary | Yes | 2 |
| 22 | Enalapril | 5mg | tab | Supplementary | Yes | 1 |
| 23 | Fluconazole | 200mg | cap/tab | Supplementary | Yes | 1 |
| 24 | Fluoxetine | 20 mg | cap/tab | Supplementary | Yes | 1 |
| 25 | Fluphenazine Decanoate | 25mg/ml | ampule | Supplementary | Yes | 2 |
| 26 | Furosemide | 40 mg | cap/tab | Supplementary | Yes | 1 |
| 27 | Glibenclamide | 5mg | cap/tab | Supplementary | Yes | 1 |
| 28 | Gliclazide | 80 mg | cap/tab | Supplementary | Yes | 2 |
| 29 | Hydrochlorothiazide | 25mg | cap/tab | Supplementary | Yes | 1 |
| 30 | Indinavir | 400mg | cap | Supplementary | Yes | 3 |
| 31 | Insulin Isophane (NPH) | 100 IU/ml | vial | Supplementary | Yes | 1 |
| 32 | Insulin Neutral Soluble (Regular) | 100 IU/ml | vial | Supplementary | Yes | 1 |
| 33 | Losartan | 50mg | cap/tab | Supplementary | Yes | 2 |
| 34 | Lovastatin | 20mg | tab | Supplementary | No | 1 |
| 35 | Metformin | 500 mg | cap/tab | Global | Yes | 1 |
| 36 | Methyldopa | 250mg | tab | Supplementary | Yes | 1 |
| 37 | Metronidazole | 400 mg | cap/tab | Supplementary | yes | 1 |
| 38 | Nevirapine | 200mg | tab | Supplementary | yes | 2 |
| 39 | Nifedipine Retard | 20 mg | tab | Supplementary | no | 2 |
| 40 | Omeprazole | 20 mg | cap/tab | Global | yes | 1 |
| 41 | Omeprazole (10) | 10mg | cap/tab | Supplementary | yes | 1 |
| 42 | Paracetamol suspension | 24 mg/ml | millilitre | Global | Yes | 1 |
| 43 | Phenytoin | 100mg | tab | Supplementary | Yes | 1 |
| 44 | Propranolol | 40mg | tab | Supplementary | Yes | 1 |
| 45 | Pyrimethamine with sulfadoxine | (25+500) mg | tab | Supplementary | Yes | 2 |
| 46 | Ranitidine | 150 mg | cap/tab | Supplementary | Yes | 1 |
| 47 | Salbutamol inhaler | 100 mcg/dose | dose | Global | Yes | 1 |
| 48 | Simvastatin | 20 mg | cap/tab | Global | Yes | 2 |
| 49 | Spironolactone | 100mg | tab | Supplementary | Yes | 1 |
| 50 | Zidovudine | 100mg | cap | Supplementary | Yes | 2 |

*1: All three levels i.e. Primary, Secondary and Tertiary, 2: Both Tertiary and Secondary levels and 3: Tertiary level only.

**Table B.** Availability of All and Essential Medicines, OB and LPG, at Public and Private Facilities

|  | Public Sector (n=16 outlets) | | | | Private Sector (n=16 outlets) | | | |
| --- | --- | --- | --- | --- | --- | --- | --- | --- |
|  | **All medicines**  **(n=50)** | | **NEML medicines only (n=46)** | | **All medicines**  **(n=50)** | | **NEML medicines only (n=46)** | |
|  | **OB** | **LPG** | **OB** | **LPG** | **OB** | **LPG** | **OB** | **LPG** |
| Mean Availability (±SD) | 6.8%  (11.2%) | 35.3%  (26.7%) | 7.1%  (11.6%) | 35.6%  (26.4%) | 55.0%  (31.1%) | 20.3%  (14.4%) | 55.3%  (31.0%) | 20.7%  (14.6%) |

**Table C.** Availability of Medicines at various Levels of Health Care (Public Sector).

|  | Primary level  (n=2 outlets) | | Secondary level (n=13 outlets) | | | | Tertiary level  (n=1 outlet) | | | |
| --- | --- | --- | --- | --- | --- | --- | --- | --- | --- | --- |
|  | **All medicines**  **(n=50)** | | | | **All medicines**  **(n=50)** | | | | **All medicines**  **(n=50)** | |
|  | **OB** | **LPG** | | **OB** | | **LPG** | | **OB** | | **LPG** |
| Mean Availability  (SD) | 0%  (0%) | 25.8%  (44.5%) | | 7.1%  (11.2%) | | 35.2%  (27.5%) | | 12.0%  (32.8%) | | 50.0%  (50.5%) |
|  | **EML medicines**  **(n=46)** | | | | **EML medicines**  **(n=46)** | | | | **EML medicines**  **(n=46)** | |
|  | **OB** | **LPG** | | **OB** | | **LPG** | | **OB** | | **LPG** |
| Mean Availability  (SD) | 0%  (0%) | 26.7%  (45%) | | 7.7%  (11.5%) | | 35.3%  (27.3%) | | 10.9%  (31.5%) | | 50.0%  (50.6%) |

**Table D.** Individual Medicines Availability in Both Public and Private Sectors.

| Individual Medicines Availability in Outlets | | | | |
| --- | --- | --- | --- | --- |
| Medicine Name | **Brand** | | **Lowest Price** | |
|  | **Public (n=16)** | **Private (n=16)** | **Public (n=16)** | **Private (n=16)** |
| Acetylsalicylic Acid | 0.0% | 25.0% | 56.3% | 25.0% |
| Aciclovir | 0.0% | 50.0% | 25.0% | 37.5% |
| Amiodarone | 9.1% | 62.5% | 18.2% | 6.3% |
| Amitriptyline | 0.0% | 31.3% | 0.0% | 25.0% |
| Amlodipine | 6.3% | 75.0% | 62.5% | 31.3% |
| Amoxicillin | 43.8% | 93.8% | 18.8% | 25.0% |
| Amoxicillin (250) | 18.8% | 100.0% | 12.5% | 31.3% |
| Atenolol | 12.5% | 87.5% | 81.3% | 43.8% |
| Atorvastatin | 9.1% | 50.0% | 18.2% | 25.0% |
| Azithromycin | 0.0% | 25.0% | 25.0% | 31.3% |
| Beclometasone inhaler | 0.0% | 6.3% | 36.4% | 12.5% |
| Bisoprolol | 6.3% | 87.5% | 18.8% | 25.0% |
| Captopril | 0.0% | 81.3% | 81.8% | 25.0% |
| Carbamazepine | 12.5% | 87.5% | 56.3% | 25.0% |
| Ceftriaxone injection | 18.2% | 68.8% | 81.8% | 31.3% |
| Ciprofloxacin | 0.0% | 81.3% | 100.0% | 37.5% |
| Clarithromycin | 0.0% | 87.5% | 45.5% | 25.0% |
| Co-trimoxazole suspension | 6.3% | 31.3% | 62.5% | 6.3% |
| Diazepam | 6.3% | 62.5% | 12.5% | 0.0% |
| Diclofenac | 54.5% | 50.0% | 36.4% | 6.3% |
| Digoxin | 0.0% | 62.5% | 36.4% | 0.0% |
| Enalapril | 0.0% | 68.8% | 62.5% | 18.8% |
| Fluconazole | 0.0% | 37.5% | 18.8% | 25.0% |
| Fluoxetine | 0.0% | 43.8% | 12.5% | 43.8% |
| Fluphenazine Decanoate | 0.0% | 0.0% | 0.0% | 12.5% |
| Furosemide | 12.5% | 93.8% | 50.0% | 6.3% |
| Glibenclamide | 18.8% | 68.8% | 56.3% | 12.5% |
| Gliclazide | 0.0% | 75.0% | 0.0% | 18.8% |
| Hydrochlorothiazide | 0.0% | 0.0% | 0.0% | 12.5% |
| Indinavir | 0.0% | 0.0% | 0.0% | 0.0% |
| Insulin Isophane (NPH) | 0.0% | 75.0% | 50.0% | 25.0% |
| Insulin Neutral Soluble (Regular) | 0.0% | 68.8% | 50.0% | 25.0% |
| Losartan | 9.1% | 43.8% | 18.2% | 43.8% |
| Lovastatin | 0.0% | 0.0% | 6.3% | 0.0% |
| Metformin | 25.0% | 81.3% | 56.3% | 31.3% |
| Methyldopa | 12.5% | 81.3% | 25.0% | 0.0% |
| Metronidazole | 25.0% | 93.8% | 56.3% | 37.5% |
| Nevirapine | 0.0% | 0.0% | 9.1% | 0.0% |
| Nifedipine Retard | 0.0% | 75.0% | 18.2% | 12.5% |
| Omeprazole | 6.3% | 56.3% | 75.0% | 37.5% |
| Omeprazole (10) | 0.0% | 12.5% | 43.8% | 18.8% |
| Paracetamol suspension | 6.3% | 68.8% | 87.5% | 25.0% |
| Phenytoin | 0.0% | 6.3% | 12.5% | 6.3% |
| Propranolol | 12.5% | 68.8% | 12.5% | 6.3% |
| Pyrimethamine with sulfadoxine | 0.0% | 75.0% | 18.2% | 6.3% |
| Ranitidine | 0.0% | 81.3% | 31.3% | 25.0% |
| Salbutamol inhaler | 0.0% | 81.3% | 56.3% | 31.3% |
| Simvastatin | 0.0% | 25.0% | 27.3% | 56.3% |
| Spironolactone | 6.3% | 62.5% | 25.0% | 0.0% |
| Zidovudine | 0.0% | 0.0% | 0.0% | 0.0% |

**Table E.** Median Price Ratio (MPR) to Reference Price; MSH, 2015 in Private Sector

| Median Price Ratio (MPR) to reference Price (MSH, 2015) | | | | | |
| --- | --- | --- | --- | --- | --- |
| Reference Price | **Sector** | **Type and no. of medicines** | **Median MPR**  **(25%-75% IQR)** | **Minimum MPR** | **Maximum MPR** |
| MSH, 2015 | **Private** | **OB (n=41)** | 2.45  (1.48-5.46) | 0.58 | 60.63 |
|  |  | **LPG(n=27)** | 1.36  (0.93-2.63) | 0.42 | 19.96 |

**Table F.** Individual Medicine Price Ratios for surveyed medicines in Private Sector.

| **No.** | **Medicine Name** | **Medicine Type** | **Median Price Ratio (MPR)** | **25%ile** | **75%ile** | **Min** | **Max** |
| --- | --- | --- | --- | --- | --- | --- | --- |
| 1 | Acetylsalicylic Acid | Brand | 0.76 | 0.71 | 0.78 | 0.65 | 0.78 |
| 1 | Acetylsalicylic Acid | Lowest Price | 0.66 | 0.61 | 0.72 | 0.61 | 0.72 |
| 2 | Aciclovir | Brand | 20.21 | 20.21 | 20.21 | 20.20 | 20.21 |
| 2 | Aciclovir | Lowest Price | 3.58 | 3.49 | 3.58 | 3.13 | 3.61 |
| 3 | Amiodarone | Brand | 2.24 | 2.23 | 2.24 | 1.94 | 2.26 |
| 3 | Amiodarone | Lowest Price | 0.84 | 0.84 | 0.84 | 0.84 | 0.84 |
| 4 | Amitriptyline | Brand | 1.72 | 1.37 | 1.72 | 1.26 | 1.72 |
| 4 | Amitriptyline | Lowest Price | 1.37 | 1.31 | 1.42 | 1.15 | 1.59 |
| 5 | Amlodipine | Brand | 7.28 | 6.93 | 7.28 | 4.88 | 7.32 |
| 5 | Amlodipine | Lowest Price | 1.10 | 1.07 | 1.83 | 0.72 | 2.44 |
| 6 | Amoxicillin | Brand | 2.77 | 2.62 | 2.77 | 1.61 | 2.89 |
| 6 | Amoxicillin | Lowest Price | 1.86 | 1.25 | 2.39 | 0.96 | 2.41 |
| 7 | Amoxicillin (250) | Brand | 2.15 | 2.11 | 2.15 | 1.81 | 3.31 |
| 7 | Amoxicillin (250) | Lowest Price | 1.81 | 1.75 | 1.87 | 1.21 | 1.87 |
| 8 | Atenolol | Brand | 5.58 | 5.49 | 5.58 | 5.41 | 6.51 |
| 8 | Atenolol | Lowest Price | 1.85 | 1.80 | 1.85 | 1.80 | 2.70 |
| 9 | Atorvastatin | Brand | 18.11 | 18.11 | 18.11 | 18.11 | 18.21 |
| 9 | Atorvastatin | Lowest Price | 3.36 | 3.14 | 3.61 | 3.14 | 3.68 |
| 10 | Azithromycin | Brand | 1.70 | 1.49 | 1.91 | 1.49 | 1.91 |
| 10 | Azithromycin | Lowest Price | 1.70 | 1.06 | 1.95 | 0.84 | 1.95 |
| 11 | Beclometasone inhaler | Brand | 1.23 | 1.23 | 1.23 | 1.23 | 1.23 |
| 11 | Beclometasone inhaler | Lowest Price | 1.16 | 1.14 | 1.17 | 1.13 | 1.18 |
| 12 | Bisoprolol | Brand | 1.19 | 1.19 | 1.20 | 1.16 | 1.21 |
| 12 | Bisoprolol | Lowest Price | 0.69 | 0.66 | 0.75 | 0.66 | 0.85 |
| 13 | Captopril | Brand | 3.00 | 3.00 | 3.02 | 2.40 | 3.14 |
| 13 | Captopril | Lowest Price | 2.55 | 2.33 | 2.60 | 1.69 | 2.74 |
| 14 | Carbamazepine | Brand | 2.56 | 2.55 | 2.56 | 2.55 | 2.61 |
| 14 | Carbamazepine | Lowest Price | 1.72 | 1.64 | 1.73 | 1.41 | 1.75 |
| 15 | Ceftriaxone injection | Brand | 16.28 | 6.30 | 16.28 | 5.57 | 16.28 |
| 15 | Ceftriaxone injection | Lowest Price | 6.78 | 6.06 | 9.42 | 4.85 | 9.42 |
| 16 | Ciprofloxacin | Brand | 13.03 | 7.50 | 13.21 | 7.24 | 13.23 |
| 16 | Ciprofloxacin | Lowest Price | 2.84 | 2.65 | 3.76 | 1.29 | 5.43 |
| 17 | Clarithromycin | Brand | 2.48 | 2.46 | 2.49 | 2.45 | 2.50 |
| 17 | Clarithromycin | Lowest Price | 1.36 | 1.22 | 1.51 | 1.13 | 1.61 |
| 18 | Co-trimoxazole suspension | Brand | 0.58 | 0.58 | 0.58 | 0.50 | 0.78 |
| 18 | Co-trimoxazole suspension | Lowest Price | 0.42 | 0.42 | 0.42 | 0.42 | 0.42 |
| 19 | Diazepam | Brand | 2.01 | 2.01 | 2.01 | 2.01 | 3.01 |
| 19 | Diazepam | Lowest Price |  |  |  |  |  |
| 20 | Diclofenac | Brand | 12.05 | 10.77 | 12.35 | 7.71 | 17.14 |
| 20 | Diclofenac | Lowest Price | 7.50 | 7.50 | 7.50 | 7.50 | 7.50 |
| 21 | Digoxin | Brand | 2.41 | 2.03 | 2.41 | 1.91 | 2.41 |
| 21 | Digoxin | Lowest Price |  |  |  |  |  |
| 22 | Enalapril | Brand | 5.42 | 5.42 | 5.56 | 5.24 | 8.21 |
| 22 | Enalapril | Lowest Price | 1.90 | 1.88 | 2.44 | 1.85 | 2.99 |
| 23 | Fluconazole | Brand | 60.63 | 60.63 | 60.64 | 60.63 | 60.64 |
| 23 | Fluconazole | Lowest Price | 19.96 | 17.42 | 29.63 | 13.72 | 54.73 |
| 24 | Fluoxetine | Brand | 9.71 | 9.71 | 9.71 | 9.62 | 9.71 |
| 24 | Fluoxetine | Lowest Price | 3.09 | 1.06 | 3.11 | 0.77 | 3.52 |
| 25 | Fluphenazine Decanoate | Brand |  |  |  |  |  |
| 25 | Fluphenazine Decanoate | Lowest Price | 1.05 | 1.01 | 1.08 | 0.97 | 1.12 |
| 26 | Furosemide | Brand | 2.99 | 2.99 | 3.00 | 1.74 | 3.16 |
| 26 | Furosemide | Lowest Price | 2.99 | 2.99 | 2.99 | 2.99 | 2.99 |
| 27 | Glibenclamide | Brand | 2.86 | 2.86 | 2.86 | 2.54 | 2.88 |
| 27 | Glibenclamide | Lowest Price | 2.66 | 2.56 | 2.75 | 2.47 | 2.84 |
| 28 | Gliclazide | Brand | 1.50 | 1.49 | 1.51 | 1.39 | 1.51 |
| 28 | Gliclazide | Lowest Price | 0.94 | 0.90 | 0.94 | 0.86 | 0.94 |
| 29 | Hydrochlorothiazide | Brand |  |  |  |  |  |
| 29 | Hydrochlorothiazide | Lowest Price | 1.58 | 1.53 | 1.63 | 1.48 | 1.68 |
| 30 | Indinavir | Brand |  |  |  |  |  |
| 30 | Indinavir | Lowest Price |  |  |  |  |  |
| 31 | Insulin Isophane (NPH) | Brand | 1.12 | 1.12 | 1.12 | 1.01 | 1.12 |
| 31 | Insulin Isophane (NPH) | Lowest Price | 0.83 | 0.81 | 0.86 | 0.81 | 0.86 |
| 32 | Insulin Neutral Soluble (Regular) | Brand | 1.05 | 1.05 | 1.05 | 0.99 | 1.05 |
| 32 | Insulin Neutral Soluble (Regular) | Lowest Price | 0.78 | 0.76 | 0.81 | 0.76 | 0.81 |
| 33 | Losartan | Brand | 4.33 | 4.33 | 4.33 | 4.33 | 4.36 |
| 33 | Losartan | Lowest Price | 0.92 | 0.84 | 1.01 | 0.25 | 1.24 |
| 34 | Lovastatin | Brand |  |  |  |  |  |
| 34 | Lovastatin | Lowest Price |  |  |  |  |  |
| 35 | Metformin | Brand | 0.99 | 0.96 | 0.99 | 0.96 | 0.99 |
| 35 | Metformin | Lowest Price | 0.96 | 0.96 | 0.96 | 0.96 | 0.99 |
| 36 | Methyldopa | Brand | 1.90 | 1.88 | 1.90 | 1.79 | 1.93 |
| 36 | Methyldopa | Lowest Price |  |  |  |  |  |
| 37 | Metronidazole | Brand | 1.28 | 1.25 | 1.38 | 1.23 | 2.57 |
| 37 | Metronidazole | Lowest Price | 1.23 | 1.07 | 1.27 | 0.82 | 1.28 |
| 38 | Nevirapine | Brand |  |  |  |  |  |
| 38 | Nevirapine | Lowest Price |  |  |  |  |  |
| 39 | Nifedipine Retard | Brand | 2.70 | 2.70 | 2.85 | 2.69 | 4.04 |
| 39 | Nifedipine Retard | Lowest Price | 1.26 | 1.00 | 1.52 | 0.74 | 1.78 |
| 40 | Omeprazole | Brand | 34.04 | 33.99 | 34.07 | 33.61 | 34.09 |
| 40 | Omeprazole | Lowest Price | 10.50 | 9.76 | 11.78 | 4.64 | 12.65 |
| 41 | Omeprazole (10) | Brand | 2.20 | 1.38 | 3.02 | 0.55 | 3.85 |
| 41 | Omeprazole (10) | Lowest Price | 0.94 | 0.74 | 1.16 | 0.55 | 1.38 |
| 42 | Paracetamol suspension | Brand | 1.58 | 1.58 | 1.59 | 1.54 | 1.59 |
| 42 | Paracetamol suspension | Lowest Price | 0.87 | 0.83 | 1.02 | 0.76 | 1.41 |
| 43 | Phenytoin | Brand | 4.82 | 4.82 | 4.82 | 4.82 | 4.82 |
| 43 | Phenytoin | Lowest Price | 0.52 | 0.52 | 0.52 | 0.52 | 0.52 |
| 44 | Propranolol | Brand | 2.17 | 2.03 | 4.30 | 1.82 | 4.36 |
| 44 | Propranolol | Lowest Price | 0.92 | 0.92 | 0.92 | 0.92 | 0.92 |
| 45 | Pyrimethamine with sulfadoxine | Brand | 1.42 | 1.41 | 1.42 | 1.34 | 1.46 |
| 45 | Pyrimethamine with sulfadoxine | Lowest Price | 1.21 | 1.21 | 1.21 | 1.21 | 1.21 |
| 46 | Ranitidine | Brand | 3.69 | 3.69 | 3.69 | 3.65 | 4.19 |
| 46 | Ranitidine | Lowest Price | 2.91 | 2.58 | 3.24 | 1.66 | 4.19 |
| 47 | Salbutamol inhaler | Brand | 1.05 | 1.05 | 1.05 | 1.05 | 1.57 |
| 47 | Salbutamol inhaler | Lowest Price | 0.67 | 0.67 | 0.67 | 0.67 | 0.91 |
| 48 | Simvastatin | Brand | 12.33 | 12.32 | 12.34 | 12.31 | 12.34 |
| 48 | Simvastatin | Lowest Price | 1.56 | 1.56 | 6.19 | 1.56 | 6.19 |
| 49 | Spironolactone | Brand | 0.84 | 0.84 | 0.84 | 0.81 | 0.86 |
| 49 | Spironolactone | Lowest Price |  |  |  |  |  |
| 50 | Zidovudine | Brand |  |  |  |  |  |
| 50 | Zidovudine | Lowest Price |  |  |  |  |  |
| (Blank if not found in any outlet) | | | | | | | |
